# HIV inhibits endothelial reverse cholesterol transport through impacting subcellular Caveolin-1 trafficking

**DOI:** 10.1186/s12977-015-0188-y

**Published:** 2015-07-15

**Authors:** Shanshan Lin, Peter E Nadeau, Ayalew Mergia

**Affiliations:** Department of Infectious Diseases and Pathology, University of Florida, Gainesville, FL 32611 USA

**Keywords:** HIV, Nef, Caveolin 1, Endothelial cells

## Abstract

**Background:**

Human immunodeficiency virus (HIV) infection leads to decreased reverse cholesterol transport (RCT) in macrophages, and Nef mediated down-regulation and redistribution of ATP-binding cassette transporter A1 (ABCA1) are identified as key factors for this effect. This may partially explain the increased risk of atherosclerosis in HIV infected individuals. Since endothelial dysfunction is key in the initial stages of atherosclerosis, we sought to determine whether RCT was affected in human aortic endothelial cells (HAECs).

**Results:**

We found that apoA-I does not significantly stimulate cholesterol efflux in HAECs while cholesterol efflux to high-density lipoprotein (HDL) was dramatically reduced in HAECs co-cultured with HIV infected cells. Studies with wild type and Nef defective HIV revealed no significant differences suggesting that multiple factors are working perhaps in concert with Nef to affect cholesterol efflux to HDL from HAECs. Interestingly, treating HAECs with recombinant Nef showed similar effect in HDL mediated cholesterol efflux as observed in HAECs co-cultured with HIV infected cells. Using a detergent-free based subcellular fractionation approach, we demonstrated that exposure of HAECs to HIV infected cells or Nef alone disrupts caveolin 1 (Cav-1) subcellular trafficking upon HDL stimulation. Moreover, Nef significantly enhanced tyrosine 14 phosphorylation of Cav-1 which may have an impact on recycling of Cav-1 and caveolae.

**Conclusion:**

These results suggest that HIV interferes with cholesterol efflux by HDL in HAECs through the disruption of Cav-1s’ cellular distribution and that multiple factors are involved, possibly including Nef, for the inhibition of HDL mediated cholesterol efflux and alteration of cellular distribution of Cav-1.

**Electronic supplementary material:**

The online version of this article (doi:10.1186/s12977-015-0188-y) contains supplementary material, which is available to authorized users.

## Background

Human immunodeficiency virus (HIV) infection is associated with high cardiac risks. An accumulating body of evidence suggests that HIV infection leads to accelerated atherosclerosis [1, 2]. Macrophages, smooth muscle cells (SMCs) and vascular endothelial cells are prominent cell types involved in the progression of atherosclerosis. Key features for atherosclerosis include the accumulation of cholesterol in macrophages and SMCs leading to foam cell formation, and vascular endothelial cell dysfunction, which is considered an early marker for atherosclerosis [3–5]. HIV has been shown to infect human arterial SMCs, and HIV p24 protein has been detected in SMCs from tissue sections of human atherosclerotic plaques obtained from HIV-infected individuals [6]. HIV infection can change endothelial cell function as well as the microenvironment that influences endothelium function [7–9]. Studies in both animal and in vitro models reveal a correlation of endothelial cell dysfunction with HIV envelope gp120, Nef, Tat, and matrix p17 [10–14]. These changes include enhanced expression of cell adhesion molecules, increased permeability of endothelial cells, stimulation of cytokine secretion, endothelial cell proliferation, and apoptosis [10–14]. Furthermore, HIV infection leads to impaired ATP-binding cassette transporter A1 (ABCA1)-dependent cholesterol efflux from human macrophages, which is mediated by Nef induced post-transcriptional down-regulation as well as redistribution of ABCA1 [15]. HIV positive foam cells are present in atherosclerotic plaques of HIV infected patients [15]. Nef treated mice have significantly increased amounts of lipid laden macrophages [16]. These results suggest that direct infection of human macrophages and arterial SMCs by HIV, as well as HIV induced endothelial cell dysfunction are involved in a potential mechanism in a multifactorial paradigm to explain atherosclerosis progression during HIV infection.

Reverse cholesterol transport (RCT) and cholesterol efflux is a pathway to transport accumulated cholesterol from vessel walls to the liver for excretion. By reducing cholesterol from vessel walls, RCT may affect atherosclerosis progression. High-density lipoprotein (HDL) is the main acceptor for cholesterol efflux from cells and considered a protector against atherosclerosis because of its role in RCT [17, 18]. There is substantial information on the influence of HIV infection on macrophage RCT, while the impact on endothelial cell RCT as well as the potential effects on endothelial cell function during HIV infection is not clearly known.

Caveolin-1 (Cav-1), an integral membrane protein of 21- to 24-kDa size, is a major structural component of caveolae and it binds to cholesterol [19]. This molecule was first identified as a major tyrosine-phosphorylated substrate of v-src [20] and is involved in multiple cellular functions including signal transduction, cholesterol trafficking and efflux, and endocytosis and transcytosis processes [21, 22]. Cav-1 is particularly abundant in endothelial cells and is crucial for the function of these cells. In endothelial cells, Cav-1 and caveolae may play a proatherogenic role. Cav-1 and caveolae promote transcytosis of low-density lipoprotein (LDL)-cholesterol particles from the blood to sub-endothelial spaces [23]. HDL co-localizes with Cav-1 on the cell surface of cholesterol-loaded endothelial cells, and as a consequence, caveolae act as major platforms to facilitate the transport of excess cholesterol to HDL on aortic endothelial cell surfaces [24].

The relationship between HIV and host factors determines the modulation of various cellular functions and replication of virus within an infected individual. There is limited information on the relationship of HIV infection and Cav-1. Recently, binding of Cav-1 to HIV envelope in the lipid rafts has shown to inhibit cell fusion and subsequently blocks envelope mediated bystander killing [25, 26]. Cav-1 expression is induced significantly in macrophages through a Tat mediated signaling pathway leading to the suppression of HIV replication [27]. In macrophages, Cav-1 overexpression restores Nef mediated impairment of cholesterol efflux to apoA-I [28]. Cav-1 within endothelial cells can act as a proatherogenic protein [29, 30], however, the potential role of Cav-1 in regulating lipid metabolism in endothelial cells and the relative importance of Cav-1 in the regulation of endothelial function under HIV infection is not known. To gain insight into the influence of HIV on lipid metabolism in endothelial cells we evaluated HDL mediated cholesterol efflux and correlated with the dynamic distribution of Cav-1. In addition, we show that recombinant Nef affects the redistribution of Cav-1 in cholesterol loaded endothelial cells upon HDL stimulation perhaps by inducing phosphorylation of Cav-1.

## Results

### HIV impairs cholesterol efflux by HDL in endothelial cells

The endothelium plays an essential role in cardiovascular health; therefore, endothelial dysfunction is a critical early element in the pathogenesis of atherosclerosis which contributes to plaque initiation and progression [4, 18]. Human aortic endothelial cells (HAECs) also have direct contact with circulating HIV infected cells as well as released viral proteins [31], and we therefore investigated the influence of HIV infection on endothelial cholesterol homeostasis by monitoring cholesterol efflux in HAECs. We first examined lipid free apoA-I mediated cholesterol efflux in HAECs co-cultured with HIV infected cells. Our results showed that unlike human macrophages apoA-I did not significantly stimulate cholesterol efflux in HAECs (Additional file 1: Figure S1). This may be due to weak expression of ABCA-1 in the HAECs [32, 33]. The inefficient apoA-I mediated cholesterol efflux in HAECs was monitored with cells activated with liver X receptor (LXR) agonist TO-901317 to stimulate ABCA1 expression and cholesterol efflux. Our results showed that after TO-901317 treatment apo-AI did not significantly promote cholesterol efflux (P ˃ 0.05) (Additional file 1: Figure S1). These results correlate with low expression levels of ABCA1 in HAECs and TO-901317 treatment (3 µM) barely elevated endogenous ABCA1 expression in HAECs (Additional file 1: Figure S1) thus confirming that apoA-I does not significantly promote cholesterol efflux in HAECs, which is consistent with Liao et al. previous observations [34].

**Figure 1:**
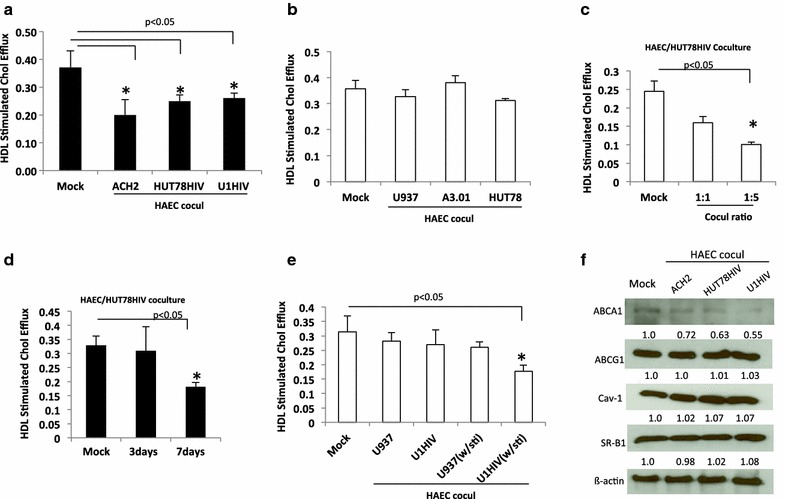
HIV impairs HDL mediated cholesterol efflux from HAECs. **a** HAECs co-cultured with HIV infected ACH2, U1HIV and HUT-78HIV, or **b** uninfected parental cell lines A3.01, U937 or HUT-78. HDL induced cholesterol efflux from HAEC was examined. **c** HAECs and HUT-78HIV were co-cultured with different HAEC/HUT-78HIV ratios 1:1 or 1:5 for 7 days, and cholesterol efflux measured. **d** HAECs were co-cultured with HUT-78HIV at ratio 1:3 and HDL mediated cholesterol efflux at days 3 and 7 were analyzed. **e** U1HIV and control cells U937 were pretreated with PMA (10^−8^ M) for 48 h before co-culture, and HAECs were co-cultured with those cells for 7 days and further subjected to cholesterol efflux examination. **f** The expression levels of ABCA1, ABCG1, SR-B1, and Cav-1 in HAECs. HAECs were co-cultured with HIV infected cells ACH2, HUT78HIV and U1HIV for 5 days. The expressions of ABCA1, ABCG1, SR-BI and Cav-1 HAECs were examined by Western blotting. Representative Western blots are shown. *Mock* HAECs cultured alone, *w/sti* PMA treated; *P < 0.05 compared with control cells.

We then monitored whether HDL mediated cholesterol efflux would be affected in HAECs co-cultured with HIV infected cells. HDL stimulated cholesterol efflux from HAECs after co-culture with ACH2 was markedly decreased with the reduction reaching 46% as compared to HAECs cultured alone (Figure 1a). The reduction was also evident when HAECs were co-cultured with HUT-78HIV and U1HIV showing a decrease by 32 and 30%, respectively. We observed no reduction in HDL mediated cholesterol efflux when the endothelial cells were co-cultured with uninfected parental A3.01, HUT-78 or U937 cells (Figure 1b). In addition, we compared HDL mediated cholesterol efflux in HAECs co-cultured with HIV infected cells (HUT-78HIV) in varying ratios and different co-culture time points. As shown in Figure 1c the 1:1 ratio showed no significant decrease whereas the 1:5 ratio showed a 56% decrease in HDL mediated cholesterol efflux. A ratio of HAECs:HUT-78HIV of 1:3 showed a decrease in HDL mediated cholesterol efflux of 44% at day 7, whereas no significant reduction was observed at day 3 (Figure 1d). Furthermore, HAECs were co-cultured with phorbol 12-myristate 13-acetate (PMA) stimulated and unstimulated U1HIV cells to further demonstrate that HIV affects HDL mediated cholesterol efflux of endothelial cells. When endothelial cells were cultured with PMA stimulated U1HIV cells HDL mediated cholesterol efflux was reduced by 44% (Figure 1e). There was only a slight decrease when the endothelial cells were cultured with unstimulated U1HIV cells. PMA treated control uninfected U937 had no influence on HDL mediated cholesterol efflux. Taken together these results show that HIV has an impact on HDL mediated cholesterol efflux in endothelial cells.

### Expression levels of Cav-1, ABCA1, ABCG1, and SR-B1 in endothelial cells

Cholesterol trafficking and efflux are regulated by various molecules that include two ATP-binding membrane cassette transport proteins (ABCA1 and ABCG1) cells, scavenger receptor type B class I (SR-BI), and Cav-1 [24, 33]. To reveal the mechanism underlying HIV mediated impairment of cholesterol efflux in endothelial cells we examined the expressions of Cav-1, ABCA1, SR-B1 and ABCG1 in HAECs co-cultured with HIV-infected cells or cultured alone by Western blot analysis. As shown in Figure 1f, the baseline expression of ABCA1 in HAECs is low and there is a slight decrease in ABCA1 when the endothelial cells were co-cultured with HIV-infected cells. In contrast, the abundant expression of Cav-1 is evident. However, the expression levels of Cav-1 remained similar whether endothelial cells were co-cultured with HIV infected cells or not. Moreover, the expression levels of ABCG1 and SR-BI remained the same whether the endothelial cells were co-cultured with infected cells or not. Therefore, the expression levels ABCA1, ABCG1, SR-BI and Cav-1 in HAECs co-cultured with HIV infected cells are not significantly affected.

### Cav-1 distribution in endothelial cells co-cultured with HIV infected cells

**Figure 2:**
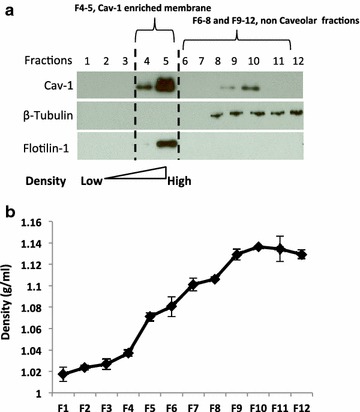
Sodium carbonate extraction and sucrose gradient isolation of caveolae membrane fractions. HAECs were homogenized and separated by a detergent-free (Na_2_CO_3_) based discontinuous sucrose density centrifugation. **a** Twelve fractions were collected from the gradient going from low to high density and were analyzed by Western blotting with antibody for specific markers of caveolae (Cav-1, Flotilin-1) β-tubulin (cytoplasmic components). **b** Density of each fraction was measured.

**Figure 3:**
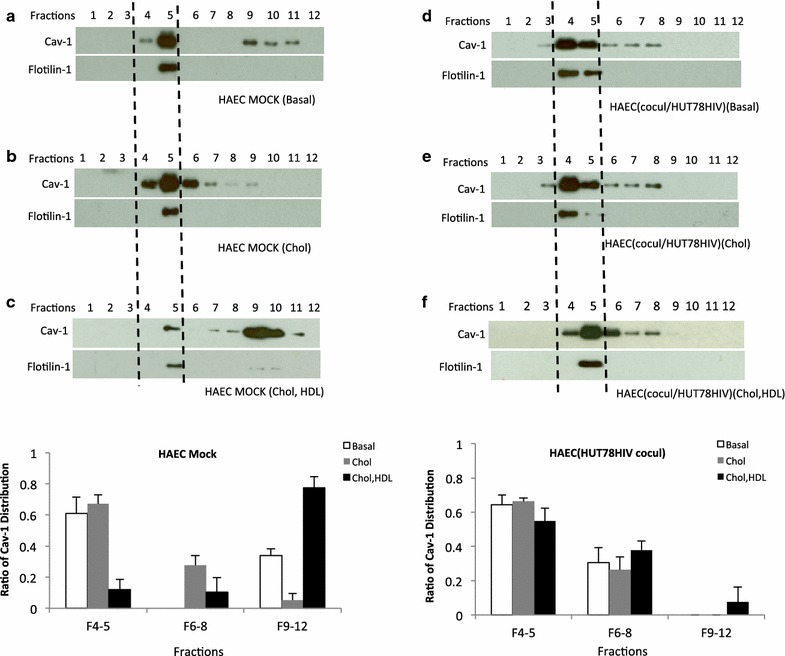
Cav-1 redistribution in HAECs co-cultured with HIV-infected cells during HDL mediated reverse cholesterol transport. HAECs were cultured in regular ECM (basal medium) (**a**), or serum free medium containing 1% fatty acid free BSA and cholesterol (40 µg/ml) for 36 h, and then further incubated in serum free medium without HDL (**b**) or with HDL (**c**) for 1–2 h. HAECs were then subjected to sucrose density gradient fractionation. Cav-1 distribution from discontinuous sucrose density gradient was examined by immunoblots. **d** HAECs were co-cultured with HUT-78HIV cells for 5 days, and HAECs were placed in regular ECM medium (cocul/HUT78HIV basal). After 5 days co-culturing with HUT-78HIV, HAECs were placed in serum free medium in the presence of cholesterol (40 µg/ml) and 1% fatty acid free BSA, and then followed incubation in medium **e** without HDL or **f** with HDL for 1–2 h. Using Na_2_CO_3_ based sucrose density gradient fractionation, 12 fractions were obtained and examined by Western blotting for Cav-1 distribution. Representative data are shown from three independent experiments. The ratio of Cav-1 distribution from mock or HUT-78HIV co-cultured HAECs in Cav-1 enriched membrane fractions 4–5 and non-caveolar fractions 6–8 and 9–12 based on three independent experiments are shown at the bottom. *Dash lines* refer to caveolae enriched membrane fractions 4 and 5.

Caveolae are the main structural platforms facilitating the transport of excess cholesterol to HDL on the aortic endothelial cell surface. Cav-1 functions as an intracellular cholesterol transporter, and cholesterol trafficking by Cav-1 is critical for HDL stimulated cholesterol efflux [24]. In addition, subcellular distribution of Cav-1 is an important parameter that influences endothelial cell functions including cholesterol homeostasis [35–37]. Therefore, Cav-1s’ subcellular localization and recycling have an effect on cholesterol efflux. To determine whether HIV can impact the cellular distribution of Cav-1 in endothelial cells, consequently impacting cholesterol efflux to HDL, HAECs were co-cultured with HIV-infected cells and subjected to sucrose gradient fractionation. The sodium carbonate-based fractionation method was used to isolate the low-density caveolae-enriched membranes. After sucrose density gradient ultra-centrifugation 12 fractions were obtained, and aliquots from each fraction were examined by Western blotting. As shown in Figure 2a Flotilin-1, a marker for caveolae enriched membranes, was observed in fraction 5, which usually presents in fractions 4 and 5. β-tubulin on the other hand, a marker for cytoplasmic components, was strictly restricted to fractions 8–12 showing no contamination among the caveolar fractions. The density of each fraction was monitored and a linear density gradient is shown in Figure 2b. In order to mimic hypercholesterolemia in HAECs cholesterol at 40 µg/ml was loaded onto the HAECs by incubating them in serum free medium containing 1% fatty acid free BSA, which significantly increased cellular cholesterol content (Additional file 2: Figure S2) compared to endothelial cells cultured in basal medium. Cav-1 was highly present in lower-density fractions 4 and 5 (caveolae fractions) with significantly lower amounts in the higher-density fractions 9–12 when endothelial cells were cultured alone in a regular endothelial cell culture basal medium (Figure 3a). In the presence of cholesterol Cav-1 distribution shifted, with increased amounts, towards the lower density caveolar fractions 4–5 (Figure 3b). When HDL was provided in cholesterol treated endothelial cells (Figure 3c), Cav-1 increased significantly in the non-caveolar fractions 9–10 with reductions in fractions 4–5 indicating an increase in recycling of Cav-1 from the membrane to the cytoplasm when HDL mediated cholesterol efflux is taking place. Interestingly, when the endothelial cells were co-cultured with HUT-78HIV cells (HIV infected) the distribution of Cav-1 shifted towards the lower density fractions 4–5 similar to that of endothelial cells cultured alone in the presence of cholesterol (Figure 3d). The distribution of Cav-1 remained in fractions 4–5, the caveolar fractions, in the endothelial cells co-cultured with HIV infected cells whether cholesterol or cholesterol/HDL was provided or not (Figure 3e, f). Furthermore, compared to the control (Figure 3c) there is a significantly lower shift of Cav-1 from fractions 4–5 into non-caveolar fractions 6–11 upon HDL stimulation (Figure 3f). These findings show that HIV alters the normal Cav-1 subcellular localization/redistribution in endothelial cells leading to a disruption in HDL mediated cholesterol homeostasis.

### The potential impact of Nef on cholesterol efflux by HDL in endothelial cells

**Figure 4:**
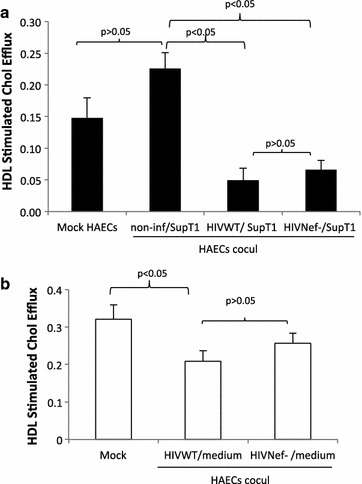
HDL mediated cholesterol efflux in HAECs co-cultured with wild type or Nef defective HIV infected cells. **a** SupT1 cells were infected with pBR43IeG-nef+ (HIV WT) or pBR43IeG-nef− (HIV Nef−). Twenty-four hours post infection cells were co-cultured with HAECs for 5 days and cholesterol efflux to HDL in HAECs was measured. **b** HAECs were treated with supernatants from uninfected, wild type, or Nef defective infected SupT1 and cholesterol efflux to HDL was analyzed 5 days post supernatant treatments.

The Nef protein has been shown to be the key molecule for the impairment of ABCA1 dependent cholesterol efflux by apoA-I of HIV infected macrophages [15]. Since our results showed that apoA-I mediated cholesterol efflux was not significantly affected in HAECs we examined the influence of Nef on HDL mediated cholesterol efflux in these cells. HAECs were co-cultured with cells infected with NL4-3 wild type (HIVWT) or Nef defective (HIVNef−) HIV strain and co-cultured with HAECs. HDL stimulated cholesterol efflux from HAECs after co-culture with HIV infected cells was significantly decreased with the reduction reaching 67 and 54% in HAECs co-cultured with both wild type and Nef defective HIV, respectively, as compared to HAECs cultured alone or 78 and 69% compared with uninfected cells (Figure 4a). Comparative analysis of HDL mediated cholesterol efflux for HAECs co-cultured with wild type and Nef defective HIV revealed that there was a minor decrease that is not significant in HDL mediated cholesterol efflux with the wild type compared to that of the Nef defective HIV. In addition, HDL mediated cholesterol efflux was measured in HAECs treated with supernatant from wild type or Nef defective HIV infected cells. As shown in Figure 4b cholesterol efflux from HAECs treated with supernatants from wild type HIV infected cells shows that there was no significant difference compared to that of Nef defective. These results taken together suggest that during co-culture multiple factors are working to affect cholesterol efflux to HDL in HAECs. If Nef plays a role then there are other factors that must have saturated HDL cholesterol efflux to not see the difference between the wild type and Nef defective HIV.

**Figure 5:**
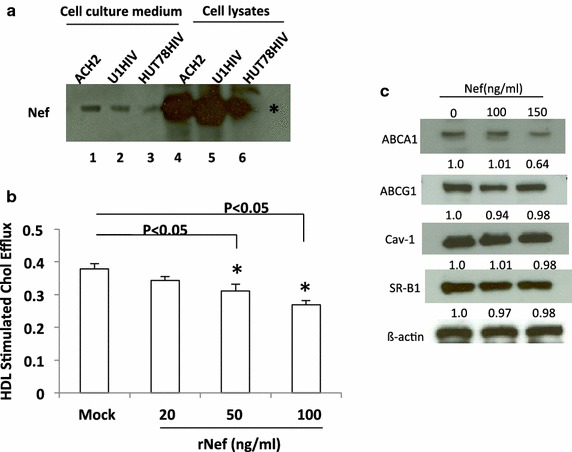
Influence of Nef on HDL mediated cholesterol efflux from HAECs. **a** Expression levels of Nef from HIV infected cells. Cell lysates and TCA and acetone precipitated culture media supernatants from infected cells were subjected to immunoblotting to detect Nef. *Lanes 1* through *3* represent released Nef from HIV infected cells and *Lanes 4* through 6 from HIV infected cell lysates. **b** HAECs were treated with recombinant Nef at indicated concentrations for 72 h, and then HDL mediated cholesterol efflux was analyzed. *P < 0.05 compare with control cells, *Mock* non-treated HAECs. **c** HAECs were exposed to recombinant Nef (0–150 ng/ml) for 48 h. The expressions of ABCA1, ABCG1, SR-BI and Cav-1 as well as eNOS from above HAECs were examined by Western blotting. Representative Western blots are shown.

To further explore the influence of Nef in HDL mediated cholesterol efflux in HAECs we determined the level of Nef released from the infected cells we used to investigate HDL cholesterol efflux and caveolin 1 re-distribution by Western blot analysis. As shown in Figure 5a there is a significant amount Nef expression in cell lysate. TCA precipitation was needed to see Nef released into the media. These results imply that the low of level Nef released from infected cells and a possible saturation of HDL mediated cholesterol efflux by other cellular factors and viral proteins may have overshadowed any potential role in cholesterol efflux by Nef. We, therefore, measured the level of HDL mediated cholesterol efflux from HAECs cultured with varying concentrations of recombinant Nef. As shown in Figure 5b a Nef dose dependent reduction of cholesterol efflux was observed with a decrease of 9, 19, or 30% when the HAECs were cultured with concentrations of 20, 50, or 100 ng/ml Nef, respectively. Therefore, Nef participates in suppressing HDL mediated cholesterol efflux in endothelial cells. The expression levels of Cav-1, ABCA1, ABCG1 and SR-BI were examined in the presence of Nef to determine whether HDL mediated cholesterol efflux suppression by Nef could be due to changes in expression of these molecules that are important in cholesterol metabolism. Similar to what was observed with co-culture of HIV infected cells the expression of ABCA1 was low with a minor effect that is not significant in Nef treated cells (Figure 5c). Consistent with HAECs co-cultured with HIV-infected cells the expression of Cav-1, ABCG1 and SR-BI remained the same whether the cells were treated with Nef or not. These results suggest that impaired cholesterol efflux to HDL from HAECs by HIV or recombinant Nef is not due to expression levels of ABCA1, ABCG1, SR-BI or Cav-1.

### Nef treatment alters Cav-1 distribution during HDL stimulation

**Figure 6:**
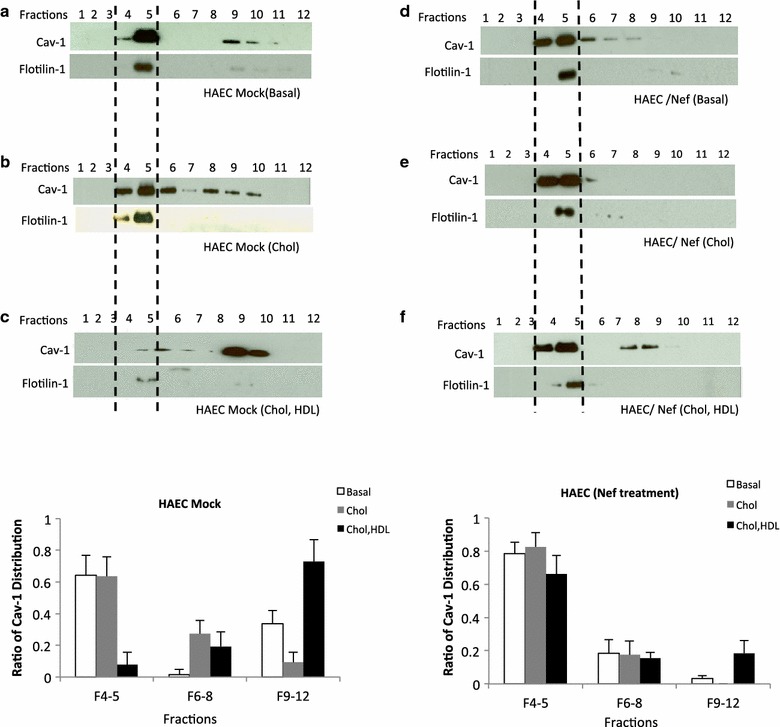
Cav-1 localization in Nef treated HAECs. **a** Cav-1 distribution was examined in HAECs cultured in regular ECM basal medium using sucrose density gradient subcellular fractionation and immunoblots. **b** Cav-1 distribution in HAECs incubated in medium containing 40 µg/ml cholesterol and 1% fatty acid free BSA. **c** Cav-1 distribution in fractions was measured in cells pre-incubated with cholesterol for 36 h followed by incubation in serum free medium containing HDL for 2 h. **d** Cav-1 localization in sucrose density fractions was examined after HAECs were incubated in regular ECM medium (basal) in presence of HIV Nef for 3 days. **e** HAECs were treated with Nef for 3 days, then incubated in medium containing 40 µg/ml cholesterol and 1% fatty acid free BSA for 36 h, followed by a 2 h incubation in serum free medium without or **f** with HDL. Ratio of Cav-1 distribution from mock or Nef treated HAECs in Cav-1 enriched membrane fractions 4–5 and non-caveolar fractions 6–8 and 9–12 is shown at the *bottom*. *Dash lines* refer to caveolae enriched membrane fractions 4 and 5. Representative data are shown from three independent experiments.

Our results show that Nef influences HDL mediated cholesterol efflux in endothelial cells, therefore, we examined whether Nef plays a role in the alteration of the cellular distribution of Cav-1 as we observed above by HIV. HAECs were cultured in the presence of Nef and the cells were then incubated with cholesterol containing medium in the absence or presence of HDL. Sucrose density gradient subcellular fractionations were subjected to immunoblot analysis to determine the levels of Cav-1 in each fraction. Untreated cells with cholesterol and HDL showed a redistribution of Cav-1 from lower (4–5) to higher (9–11) density fractions (Fig. 6a–c). In the presence of Nef Cav-1 mainly remained enriched in the caveolae fractions 4–5 similar to endothelial cells co-cultured with HIV infected cells (Figure 6d). Cav-1 was also enriched in fractions 4–5 with cholesterol loading in the presence or absence of HDL (Figure 6e, f), suggesting that Nef plays an important role in influencing Cav-1 redistribution and alteration of HDL mediated cholesterol efflux by HIV. The decrease is also more pronounced in the presence of cholesterol and HDL similar to cells co-cultured with HIV infected cells. Therefore, Nef affects the subcellular distribution of Cav-1 consequently contributing to the inhibition of HDL mediated cholesterol efflux in endothelial cells.

### Nef stimulates Cav-1 phosphorylation

**Figure 7:**
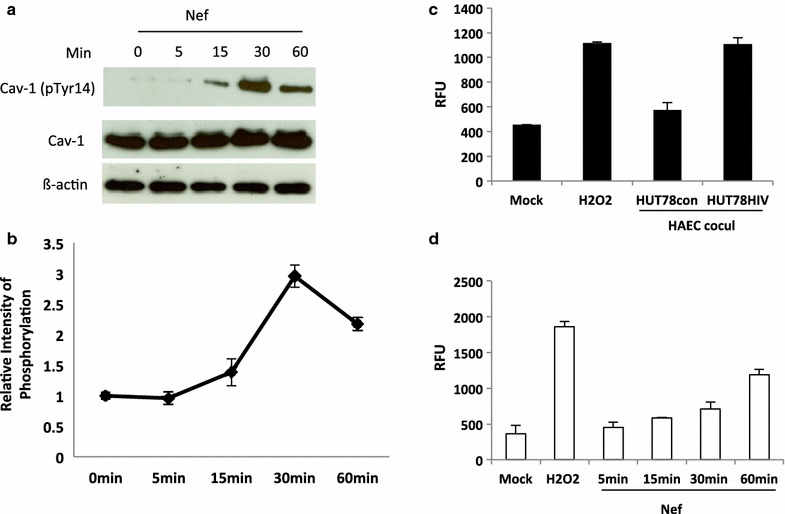
Nef induced Cav-1 phosphorylation in HAECs. **a** HAECs were grown to 80–90% confluence. After changing to fresh growth medium, HAECs were exposed to Nef for 0–60 min. Phosphorylation of Cav-1 (Tyr14) was determined by Western blotting. The blots are representative of three separate experiments. **b** Relative density of phosphorylation protein (Phos Cav-1/total Cav-1) is shown. **c** Increased ROS production in HAECs co-cultured with HIV infected HUT-78 cells at ratio of 1:5 for 3 h. **d** Increased ROS production in HAECs treated with Nef at different time points.

Src phosphorylation of Cav-1 tyrosine 14 (Tyr14) is tightly associated with the regulation of multiple endothelial functions including endocytosis, transcytosis and permeability [38–43]. In addition, cholesterol transport by caveolae also shares many features with the caveolae endocytic process and is modulated by Src activation [44, 45], thus Cav-1 Tyr14 phosphorylation can affect Cav-1 trafficking. Since Nef has been shown to influence the activity of Src kinases we speculated that the disruption of Cav-1 distribution by Nef might have to do with Nef inducing phosphorylation of Cav-1. Therefore, we examined the Tyr14 phosphorylation of Cav-1 in endothelial cells treated with Nef at different time intervals for 60 min. As shown in Figure 7a, b, Nef induced Cav-1 phosphorylation at Tyr14 at the 15 min time point reaching a maximum level at 30 min. Oxidative stress induces Tyr14 phosphorylation of Cav-1 by activating Src kinases and Nef is shown to induce oxidative stress. Thus reactive oxygen species (ROS) production was measured in endothelial cells co-cultured with HIV infected cells. As shown in Figure 7c ROS production was significantly enhanced in HAECs co-cultured with HUT-78HIV as compared to the control mock and endothelial cells co-cultured with uninfected HUT-78 cells. In addition, Nef treatment significantly activates ROS production within an hour of addition (Figure 7d). These results suggest a potential role for Nef induced Tyr14 phosphorylation of Cav-1 in the HIV mediated disruption of Cav-1 subcellular distribution and alteration of cholesterol transport to HDL. Furthermore, the phosphorylation of Cav-1 by Nef could be mediated through Nef induced ROS activation.

## Discussion

Human immunodeficiency virus regulates RCT in macrophages leading to the accumulation of cholesterol in macrophages followed by foam cell transformation, which provides the evidence for HIV related atherosclerotic diseases. However, the alteration of RCT and cholesterol efflux within vascular endothelial cells remains unsolved. Cav-1 is expressed in macrophages, SMCs and vascular endothelial cells, the three prominent cell types which are all involved in atherosclerosis. This molecule, which is particularly enriched in endothelial cells and the most prominent component of endothelial caveolae, is considered a crucial regulator for cholesterol homeostasis in vascular endothelial cells [21, 22, 29, 30, 46]. Endothelial dysfunction is considered an early marker for atherosclerosis and well-established response to cardiovascular risk [4, 5, 18, 47, 48]. In this study, we investigated cholesterol efflux in HAECs co-cultured with HIV-infected cells. Our results showed that cholesterol efflux to HDL is significantly reduced in endothelial cells when co-cultured with HIV-infected cells. There was no significant difference in HDL mediated cholesterol efflux in endothelial cells co-cultured with wild type or Nef defective HIV suggesting multiple factors are involved in HIV induced suppression of HDL mediated cholesterol efflux in HAECs. Furthermore, the reduction is related to impaired Cav-1 redistribution upon HDL stimulation. Interestingly, treatment of HAECs with recombinant Nef revealed a similar effect as HIV infection on cholesterol efflux and Cav-1 redistribution. In addition, Nef induced phosphorylation of Cav-1 at Tyr14 in the endothelial cells, which can influence Cav-1 redistribution. These results show that there is an HIV mediated disruption of cholesterol efflux in endothelial cells, and Cav-1 plays an important role in this process through a change in its trafficking with Nef possibly contributing to these alterations by inducing phosphorylation of Cav-1 Tyr14.

High-density lipoprotein classically functions in the RCT removing cholesterol from peripheral tissues to the bloodstream. Our results show that HIV impairs HDL mediated cholesterol efflux in endothelial cells and that Nef possibly plays a role in this impairment. Interestingly, apoA-I mediated cholesterol efflux was not significant in endothelial cells whether HIV infected cells are present or not, possibly due to low-level endogenous ABCA1 expression. The low level of ABCA1 expression is consistent with studies from others and their results also showed that HDL but not lipid-free apoA-I promotes cellular cholesterol efflux in human umbilical vascular endothelial cells and HAECs under normal conditions [32, 33]. The failure to stimulate ABCA1 dependent cholesterol efflux to apoA-I in endothelial cells in both the presence and absence of HIV shows that ABCA1 is not a major factor in the impairment of RCT from endothelial cells, which is different from previous findings in HIV infected macrophages.

Cav-1 dependent signal transduction and cholesterol trafficking is essential to maintain cellular cholesterol homeostasis. Cav-1 participates in cholesterol trafficking and co-localizes with HDL in cholesterol loaded endothelial cells, indicating caveolae are the major cellular platforms facilitating the transport of excess cholesterol to HDL on the cell surface of aortic endothelial cells [24, 33]. The impact of HIV on the trafficking of Cav-1 as well as HDL mediated cholesterol efflux in endothelial cells would then affect RCT leading to impaired endothelial function. Our results demonstrate HIV or recombinant Nef affects the redistribution of Cav-1 in cholesterol loaded endothelial cells upon HDL stimulation. Nef has been shown to present on surface of infected cells [49], and is also known to be secreted from infected cells [50–52] and exists in infected patients serum [52]. Nef can be secreted from monocytes and T cells that circulate in the blood stream. This protein is found in the blood stream at significant levels [52]. Furthermore, released Nef is shown to enter into uninfected cells inducing changes in the recipient cells [53–58]. Therefore, Nef can be released from infected cells and enter endothelial cells which then subsequently modulate Cav-1 cellular distribution altering HDL mediated cholesterol efflux. The reason we have not seen a significant difference in HAECs between wild type and Nef defective HIV could be due to a saturation effect on cholesterol efflux by other factors and a higher amount of Nef is needed to demonstrate the impact of Nef on HDL mediated cholesterol efflux. Alternatively, since Nef released from infected cells is associated with exosomes the influence on HDL mediated cholesterol efflux may not be as efficient as using recombinant Nef.

Post-transcriptional modification is important for Cav-1 function in cholesterol homeostasis. It is well documented that Src phosphorylation of Cav-1 is a requirement for activation of the caveolar transport machinery [38–42]. In fact, cholesterol-induced translocation of Cav-1 is modulated by Src activation [44]. In addition, elevated cholesterol stimulates caveolar endocytosis. This process also requires Src kinase and can be modulated by altering the balance of cholesterol and Cav-1 at the plasma membrane [45]. Src kinase induced phosphorylation of Cav-1 dependent signaling is crucial for endothelial function. Cav-1 phosphorylation at Tyr14 is required for integrin-regulated caveolae internalization [59]. Our results, for the first time, shows that Nef induces Cav-1 phosphorylation at Tyr14. This establishes a basis for a link of the association of Nef induced Cav-1 phosphorylation for increased endothelial dysfunction and atherosclerosis in HIV infection derived lipid disorders. Cav-1 phosphorylation is important in caveolae formation and internalization [59, 60]. However, further study is needed to determine whether phosphorylation of Cav-1 at Tyr14 by Nef affects endothelial caveolae dynamics, contributing to changes in Cav-1 distribution upon HDL stimulated cholesterol efflux.

## Conclusion

The present studies provide the first evidence for the regulation of endothelial cell RCT by HIV. The intracellular dynamic distribution of Cav-1 in vascular endothelial cells upon HDL stimulation was impacted in the presence of HIV infected cells or recombinant Nef. Induction of phosphorylation of Cav-1 by Nef observed may be a clue to further dissect the molecular mechanisms of these HIV induced changes in endothelial cells. Furthermore, our findings establish that HIV impairs HDL mediated cholesterol efflux in endothelial cells. Recombinant Nef also has a similar effect on HDL mediated cholesterol efflux in endothelial cells. Taken together these data show the role of Cav-1 in the regulation of RCT in HAECs by HIV which can lead to endothelial cell dysfunction and provides a novel candidate to target for modulating HIV infection related atherosclerosis and cardiovascular diseases.

## Methods

### Cell cultures

Human aortic endothelial cells (HAECs), originally isolated from human aorta, were purchased from ScienCell Research Laboratories. Recommended endothelial cell culture medium (ECM, ScienCell Research Laboratories, Carlsbad, CA, USA) was used for the culturing of HAECs. HAECs at passages 4–7 were used in all experiments. SupT1, ACH2, A3.01, HUT-78HIV, HUT-78, and U1HIV were kindly provided by the NIH AIDS Research Program. SupT1 cells were cultured in RPMI-1640 containing 10% FBS and penicillin–streptomycin (100 µg/ml). ACH2 is a cloned T-lymphocyte cell derived from A3.01 that are chronically infected with HIV, which consistently produces a low level of virus particles. ACH2 and A3.01 cells were maintained in RPMI 1640 supplemented with 10 mM HEPES, penicillin–streptomycin (100 µg/ml), 2 mM l-glutamine and 10% FBS. HUT-78HIV is a T lymphocyte cell line chronically infected with HIV-1SF2. HUT-78HIV and uninfected HUT-78 cells were grown in RPMI 1640 with penicillin–streptomycin (100 µg/ml) and 10% of FBS. U1HIV is a U937 monocytic cell line infected with HIV, which maintains low constitutive expression of virus. U1HIV and U937 cells were maintained in RPMI 1640 supplemented with penicillin–streptomycin (100 µg/ml), 2 mM l-glutamine and 10% FBS. Co-culture of HAECs with HIV-1-infected cells was performed by adding ACH2, U1HIV, or HUT-78HIV to a monolayer of HAEC in 12 well plates and subsequently incubating for 5 days. ACH2 and U1HIV cells were pre-treated with phorbol 12-myristate 13-acetate (PMA, 10^−8^ M) for 1–2 days to promote HIV replication. PMA stimulated and un-stimulated ACH2 or U1HIV were co-cultured with HAECs. Controls consisted of HAECs cultured alone or co-cultured with HIV-free parental cells A3.01, U937 and HUT-78. For subcellular fractionation, HIV-infected cells were added to HAECs at 60–70% confluency in 100-mm culture dishes and incubated for 5 days. HUT-78HIV cells were cultured for 3–4 days allowing virus to reach peak production at which point they were co-cultured with HAECs. The culture medium ratio for HAECs and HIV-infected cells is 1:1. Non-adherent cells were removed and adherent HAECs were checked by microscopy to ensure removal of HIV-infected cells before harvesting. Afterwards, HAECs were cultured in regular ECM (basal medium), or in serum free medium containing 0.1% BSA and cholesterol (40 μg/ml) for 36 h, followed by serum free medium without or with HDL (50 μg/ml) for 1–2 h. Nef treatment was carried out by growing HAECs to 70–80% confluency in 100-mm culture dishes with further incubation of the cells in the presence of recombinant Nef (100 ng/ml) for 72 h.

### Cholesterol efflux

To determine cholesterol efflux by HDL, HAECs were co-cultured with HIV-infected cells (ACH2, HUT-78HIV, U1HIV) or HIV-free parental cell lines (A3.01, HUT-78, U937) at a ratio of 1:3 for 5 days, respectively. ACH2 and U1HIV were pretreated with PMA (10^−8^ M) for 48 h before co-culture. HAECs and HUT-78HIV cells were co-cultured at ratio 1:1 or 1:5 for 7 days and at ratio 1:3 for 3 and 7 days. HAECs were also co-cultured with PMA (10^−8^ M) pre-treated (for 48 h) U1HIV or U937 at ratio of 1:3 and incubated for 7 days. HIV-infected cells were then removed and HAECs were rinsed with serum free medium and labeled with 1 μCi/ml [^3^H] cholesterol and incubated for 36–48 h. Cells were washed gently and cultured for an additional 18 h in serum free medium in the presence or absence of 50 μg/ml HDL (Biomedical Technologies Inc., Stoughton, MA, USA). HDL or mediated cholesterol efflux was determined as described previously [28]. Cholesterol efflux by HDL was measured as the total percentage of radiolabeled cholesterol appearing in the medium in the presence of HDL after subtraction of values of HDL-free medium as described previously [28]. To determine cholesterol efflux from HAECs by apoA-I, cells were labeled with 1 μCi/ml [^3^H] cholesterol for 24 h. Cells were treated or not with 3 µM LXR agonist TO-901317 (Sigma) for 18 h. After additional culture for 3, 6 or 24 h in presence or absence of 50 μg/ml apoA-l (Biomedical Technologies Inc., Stoughton, MA, USA), counts per minute (CPM) radioactivity levels in culture medium and cell lysates were measured using liquid scintillation counting. Results are cpm in medium as a percentage of cpm in medium plus cpm in cell lysates.

To determine the effect of Nef on cholesterol efflux in HAECs NL4-3 based Nef-positive (pBR43IeG-nef+) and Nef-negative (pBR43IeG-nef−) constructs were obtained from the NIH AIDS Research Program. Infectious virus were generated by calcium phosphate transfection of 293T cells [27]. SupT1 cells were infected with pBR43IeG-nef+ (HIV WT) or pBR43IeG-nef− (HIV Nef−) at a multiplicity of infection (MOI) of 0.005. Twenty-four hours post infection cells were co-cultured with HAECs at ratio 5:1 and medium ratio 1:1 and incubated further for 5 days. SupT1 cells were removed carefully and cholesterol efflux to HDL in HAECs was measured as described above. In a separate experiment, SupT1 cells were infected with HIV WT or HIV Nef- at an MOI 0.005 for 12 days. The supernatants were then harvested and clarified by 3,000 rpm centrifugation and added to HAECs culture at a medium ratio 1:1. HAEC cultures treated with supernatants from infected cells were further incubated for 5 days and cholesterol efflux to HDL was analyzed. To further explore the effect of Nef on HDL mediated cholesterol efflux in HAECs exogenous Nef was added at concentrations of 20, 50, and 100 ng/ml. Cells were incubated for 3 days and cholesterol efflux by HDL was measured as described above.

### Sodium carbonate based isolation of Cav-1 enriched membrane fractions

Human aortic endothelial cells from co-culture with HIV infected cells or Nef treated HAECs were harvested using 2 ml of ice-cold 500 mM sodium bicarbonate (Na_2_CO_3_) buffer containing 1 mM sodium orthovanadate (Na_3_VO_4_), 1 mM sodium fluoride (NaF), 44 μg/ml phenylmethylsulfonyl fluoride (PMSF), and a protease inhibitor mixture [61, 62]. After a 30-min incubation at 4°C, the cells were homogenized with 20 strokes on ice using a pre-chilled Dounce homogenizer and then sonicated with three 20-s bursts. Equal amounts of protein for each sonicated sample was adjusted to 45% sucrose by adding an equal volume of 90% sucrose in 2-(*N*-morpholino)ethanesulfonic acid (MES)-buffered saline (MBS) to give a final volume of 4 ml. The sample was placed in a Beckmann ultracentrifuge tube and overlaid with 4 ml of 35% sucrose, followed by an overlay of 4 ml of 5% sucrose, both in MBS containing 250 mM Na_2_CO_3_. The sucrose gradient was centrifuged at 39,000 rpm for 19 h at 4°C in a swing out Beckmann SW41 rotor. A total of 12 fractions, 1 ml each, from each gradient were collected from top down. Three hundred microliters of each fraction was precipitated with 10% trichloroacetic acid and washed with acetone. Each fraction was analyzed by SDS-PAGE and the protein of interest was detected.

### Determination of cell cholesterol content

Human aortic endothelial cells were incubated in regular ECM culture basal medium or in serum free medium containing cholesterol and 1% fatty acid free BSA for 36 h, and cellular cholesterol content was assayed using the Amplex Red cholesterol Assay Kit (Invitrogen, Carlsbad, CA, USA) according to the manufacturer’s protocol.

### Western blotting

Human aortic endothelial cells were extracted using lysis buffer [50 mM Tris pH 7.5,100 mM NaCl, 1 mM EDTA, 0.1% (v/v) Triton X-100, 10 mM NaF, 1 mM PMSF, and 1 mM Na3VO4] with a complete protease Inhibitor mixture (Roche Diagnostics, Indianapolis, IN, USA) and subjected to SDS-PAGE followed by Western blot with the appropriate primary antibody overnight at 4°C and horseradish peroxidase-conjugated secondary antibody for 1 h at room temperature. For determination of Nef expression, culture medium from HIV-infected cells was harvested and clarified at 3,000 rpm centrifugation. Cell culture supernatants were precipitated with trichloroacetic acid TCA (10%) and pellets were collected at 14,000 rpm centrifugation. Pellets were washed four times with ice-cold acetone and resuspended in 2× SDS loading buffer. Protein bands were detected by an ECL kit [chemiluminescent immunodetection system (Amersham, Piscataway, NJ, USA)]. Antibodies used for immunoblots were mouse anti-Phospho-Caveolin-1(pY14) (BD Biosciences, San Jose, CA, USA), rabbit anti-Caveolin-1 (Cell Signaling Technology Inc. Danvers, MA, USA), anti-Flotillin-1 (Santa Cruz Biotechnology, Santa Cruz, CA, USA), anti-ß-actin protein, anti-ß Tubulin (Sigma, St. Louis, MO, USA), anti-Nef (the NIH AIDS Research Program), mouse monoclonal anti-ABCA1, rabbit SR-B1 antibody, and ABCG1 antibody (Novus Biologicals).

### Cav-1 phosphorylation assays

Human aortic endothelial cells were grown to 90% confluency, subsequently washed twice with 1× Hank’s Balanced Salt Solution (HBSS) and replaced with fresh complete growth medium. Cells were then treated with 150 ng/ml Nef and collected at various time periods ranging from 5 to 60 min. Cells were lysed and subjected to Western blotting as described above.

### Reactive oxygen species (ROS) measurement

OxiSelect™ In Vitro ROS Assay Kit (Cellbiolabs, San Diego, CA, USA) was used to measure ROS according to the manufacturer’s protocol. Briefly, HAECs were first pretreated with 1 mM 2′,7′-dichlorodihydrofluorescein diacetate (DCFH-DA) a cell permeable probe oxidized to elicit fluorescence by ROS for 60 min at 37°C. Cells were then treated with recombinant Nef at 100 ng/ml for various time points (0–60 min), or co-cultured with HUT78HIV at a 1:5 ratio for 3 h. After Nef treatment or co-culture, the HAECs were washed and lysed and further subjected to fluorescent detection by Synergy™ HT Multi-Detection Microplate Reader **(**Bio-Tek Instruments, Inc.) at 480 nm/530 nm.

### Statistical analysis

Student’s t-test was applied to analyze the differences between sets of data. All analyses were performed with SPSS 12.0.1 for Windows, and were considered significant at P < 0.05.

## Additional files

Additional file 1: Figure S1. Apo-AI does not significantly stimulate cholesterol efflux in HAECs. (A) HAECs were loaded with cholesterol for 24 h, treated with 3 µM LXR agonist TO-901317 for 18 h and further incubated in presence or absence of apo-AI at the indicated time points. Cholesterol efflux was measured and results presented are representative of 3 independent experiments performed in triplicates. (B) Western blotting analysis of ABCA1 expression in HAECs treated with 1 µM or 3 µM LXR agonist TO-901317 treated HAECs. Additional file 2: Figure S2. Loading of cells with cholesterol by adding cholesterol in combination with BSA is reproducible. HAECs were cultured in regular endothelial cell culture medium (basal medium) or incubated in serum free medium in the presence of 40 μg/ml cholesterol and 1% fatty acid free BSA for 36 h, and cellular cholesterol content was assayed. Values are means of triplicate assays (±SD).
