# Supplementary figures and images for: HIV inhibits endothelial reverse cholesterol transport through impacting subcellular Caveolin-1 trafficking
Source: Retrovirology. 2015 Jul 15;12:62. doi: 10.1186/s12977-015-0188-y (PMC4501058; doi:10.1186/s12977-015-0188-y)

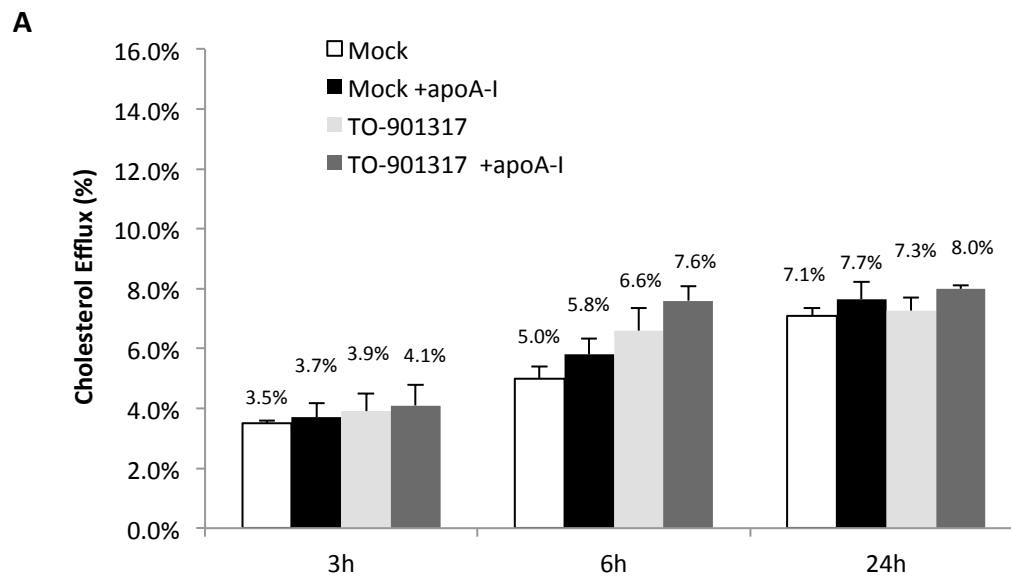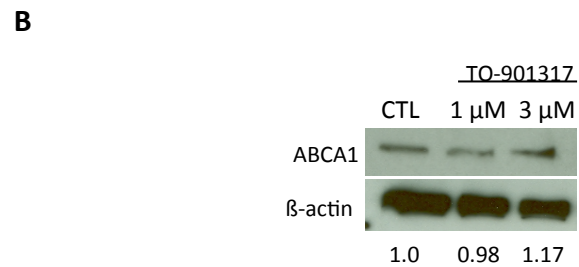

Figure S1

Supplement: Additional file 1: Figure S1. — Apo-AI does not significantly stimulate cholesterol efflux in HAECs. (A) HAECs were loaded with cholesterol for 24 h, treated with 3 µM LXR agonist TO-901317 for 18 h and further incubated in presence or absence of apo-AI at the indicated time points. Cholesterol efflux was measured and results presented are representative of 3 independent experiments performed in triplicates. (B) Western blotting analysis of ABCA1 expression in HAECs treated with 1 µM or 3 µM LXR agonist TO-901317 treated HAECs. [file 12977_2015_188_MOESM1_ESM.pdf]

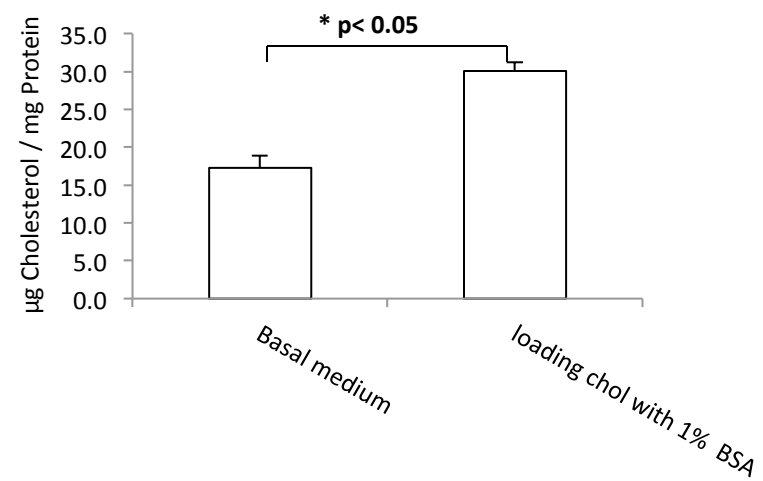

Figure S2

Supplement: Additional file 2: Figure S2. — Loading of cells with cholesterol by adding cholesterol in combination with BSA is reproducible. HAECs were cultured in regular endothelial cell culture medium (basal medium) or incubated in serum free medium in the presence of 40 μg/ml cholesterol and 1% fatty acid free BSA for 36 h, and cellular cholesterol content was assayed. Values are means of triplicate assays (±SD). [file 12977_2015_188_MOESM2_ESM.pdf]
